# Aholoa Lāna‘i!

**DOI:** 10.31372/20200503.1093

**Published:** 2020

**Authors:** May K. Kealoha

**Affiliations:** Kapiolani Community College, Hawai‘i, United States

**Keywords:** asthma, remote areas, asthma education, community participatory action

## Introduction

Asthma is a major public health concern for the state of Hawai‘i, where 10% of adults reported current asthma status in 2016 (Centers for Disease Control and Prevention, 2018). Each county reported high prevalence rates: Hawai‘i, 19.6%; Honolulu, 17.3%; Kaua‘i, 15.4%; Maui, 17.2% (Hawai‘i Health Data Warehouse, 2016). Lāna‘i is the smallest island in the State consisting of 141 square miles and a population of 3,102 (U.S. Census Bureau, 2018). Although robust asthma data exist for metropolitan Honolulu, statistics are unavailable for Lāna‘i. If data were to be extrapolated using whole ethnic populations and adult asthma rates (Hawai‘i Health Data Warehouse, 2016), 184 (13%) Filipinos and 200 (28.4%) part-Hawaiian and other Pacific Islanders may be affected by the condition.

## Problem/Significance of Topic

The island of Lāna‘i is 98% owned by Larry Ellison who employs most of the residents for tourism and environment restoration. The residents are closely knitted and live near the elementary and high school, cultural center, Maui County service departments, churches, single gas station, 3 markets, and 2 hotels. Due to Lāna‘i’s remote location and small population, health resources and specialty medical services are limited. There are 2 health clinics, 1 pharmacy, and the Lanai Community Hospital offers 24-hour urgent and limited emergency care, as well as skilled and long-term care services. Lāna‘i residents either fly or take the ferry to receive specialty care in Honolulu or Maui. Costs and complications of airfare, ground transportation, housing or hotel procurement, loss of work time, and arranging child-care for children staying on the island are overwhelming for many families (Kealoha, Sinclair, & Richardson, 2019). With median household incomes of $50,994 (U.S. Census Bureau, 2018), the cost of travel is a serious concern for families affected with asthma.

## Methods

The Aholoa Lāna‘i! project was based on a community participation model that builds on assumptions underlying community-based participatory research (CBPR) that encourages engagement and shared leadership of community members and leaders in the process of problem identification, project development, implementation, and evaluation (Kulbok, Thatcher, Park, & Meszaros, 2012). The project began by the author, a Kapi‘olani Community College nursing professor, who asked a retired Lāna‘i public health nurse (PHN) if asthma was a community concern. The retired PHN, in turn, asked a community leader to post an email announcement inviting the entire community to attend a meeting to discuss this question. The initial 1-hour meeting held at the Senior Center in August 2018 was attended by 9 community members: retired PHN, Lāna‘i PHN, Lāna‘i Elementary and High School Health Assistant, Lāna‘i Straub Clinic manager, editor of *Lanai Today*, 3 residents with asthma, and 1 resident without asthma. The attendees agreed that asthma was a serious community concern particularly due to vegetation (pine trees) and environment elements (dust and volcanic air pollution known as vog) affecting asthma. Moreover, the lack of specialty medical services required attendees/residents to travel off island for long awaited appointments (allergy, pediatric, pulmonology, diagnostic testing, etc.).

The final committee membership of 8 residents and leaders included the retired PHN, Lāna‘i PHN, Lāna‘i Elementary and High School Health Assistant, Lāna‘i Straub Clinic manager, editor of *Lanai Today*, 1 community member with asthma, Lāna‘i Community Health Center (LCHC) senior nurse practitioner, and University of Hawai‘i, Maui College, Lanai Education Center site coordinator. The nursing professor became the project coordinator. A total of 7 meetings were held from September 2018 to March 2019 for 1-hour and held at the Senior Center or Lāna‘i Public Library.

To honor the Hawaiian people and culture, the project was entitled Aholoa Lāna‘i! “Aholoa” conveys “breathing well, deeply, patiently” like a free diver or Hawaiian oli chanter. The Committee set the following goals: (1) to provide health education experiences about asthma control to affected adults and children; (2) for the general community to recognize when a person is having an asthma attack and know what to do to help; (3) to provide opportunity for Lāna‘i residents with asthma to be introduced to specialists and receive initial consultation, if desired; and (4) to expose students to health professions. The Committee planned and coordinated two community events to meet the identified goals: (1) the Lāna‘i Community Health Fair on February 22, 2019 and (2) the Asthma Care Workshop on February 23, 2019.

Each member of the Committee contributed to the planning and organization of both events. Those familiar with patients with asthma (PHN, School Assistant, health centers, and family/friends) extended personal invitations. The project coordinator designed flyers that were posted by Committee members throughout the island. The School Assistant served as the liaison to teachers to promote lung health and join the upcoming events. LCHC Community Health Workers taught and supervised the elementary children’s creation of lung health posters. The project coordinator invited vendors as listed by the Committee. The *Lāna‘i Today* editor published the events free of charge and arranged for the ILWU to donate the use of the union hall. The Maui College Lanai Education Center site coordinator and staff enlisted and supervised high school students to setup/clean the hall and to staff the registration table.

The project coordinator met with O‘ahu specialists including a pediatric pulmonologist, internist, respiratory therapist, culinary chefs, and an American Lung Association educator to plan the Asthma Workshop and arrange for transportation. She secured funding and asthma equipment through the University of Hawai‘i SEED grant, New Hope Mānoa church, and professional organizations like the Hawaii COPD Coalition, Kapi‘olani Community College Culinary and Respiratory Departments, and others

## Results

Sixteen vendors from Lāna‘i, O‘ahu, and Maui participated in the Community Health Fair including the Committee members’ respective organizations. Health education, resources, health screening (vision and spirometry), free reading glasses, prizes, and dinner were offered. School children displayed their lung health posters. Attendance exceeded expectation (100 expected/127 attended).

Although the Asthma Care Workshop attendance exceeded expectation (64 expected/60 attended), participation of those with asthma was less than expected (30 expected/17 attended). To better understand the current asthma status of the participants, those with asthma (17) completed the Asthma Control Test (ACT) that indicated 24% had controlled asthma; 76% had uncontrolled asthma; and most did not appear to have an Asthma Action Plan. Specialists provided one-to-one consultation. Participants received group asthma education about asthma triggers, medication administration, actions to take during asthma attack, asthma action plans, smoking cessation; sampled healthy smoothies, and performed spirometry testing. The 7th grade math students practiced research and math skills by designing an asthma survey. Free breakfast and lunch were served. At the recommendation of the pediatric pulmonologist, a Round Table Discussion was held during the Asthma Workshop lunch break at Lāna‘i Hospital to network with 6 island health care providers: Lāna‘i Hospital emergency room physician and Director of Nursing, Straub Clinic Medical Director, Community Health Center APRN and DNP student, and Lāna‘i PHN.

## Discussion

At the evaluation meeting, Committee members expressed their pleasure with the participation by the community in both events. According to verbal reports solicited by Committee members, the community responses were deemed to be positive. Limitations involved the lack of objective data to know whether the goals were met. Asthma control was not reached by all those with asthma but the Committee believed that participants were provided information about recognizing asthma attacks and actions to take; those with asthma were given an initial medical workup and referral for continued care with their health care provider; and students were given opportunity to observe health professionals. Unexpected benefits were recognized such as the spirometer training of 3 medical assistants that was arranged by the Clinic manager with the visiting respiratory therapist and face-to-face networking by Lāna‘i health care providers with specialists who made themselves available for continued contact.

Recommendations by the Committee involved brainstorming how to increase attendance, food to serve, practice preparing healthy foods, and provide opportunities for students to interact with specialists. At the end of the lively and camaraderie session, all of the members expressed keen interest in under-taking another community action project in the future.

## Recommendations

The Aholoa Lāna‘i! project demonstrated that many volunteers “on and off island” were eager to assist the residents of Lāna‘i. By utilizing the community participation model approach, community members and health care partners can effectively join together to bring health related services to isolated areas.

Geographic barriers are real and complex. Providing a wide range of health education and specialty services is difficult for isolated and small communities to do unaided. More effort is required by health care professionals to lobby the federal, state, and county governments to deliver comprehensive medical services and educational and supportive programs for families living on all islands. Services may be extended by telehealth, mentoring/instruction on site to key personnel, and devising a network for island health care professionals to easily seek and receive assistance and consultation regularly.

## Acknowledgments

Volunteer contribution by Lāna‘i Leadership Committee, Lāna‘i Community, Lāna‘i School, Public Health Nursing, Lāna‘i Community Health Center, Lāna‘i Straub Clinic, UH Maui College, Lanai Education Center, *Lanai Today*, New Hope Mānoa, Kapi‘olani Community College—Culinary, Nursing, Respiratory Departments, American Lung Association, respiratory therapist, and physicians.

## Funding

Funding was provided in part by University of Hawai‘i SEED Inclusion, Diversity, Equity, Access and Success (IDEAS) grant.
